# “*Maybe his blood is still strong*”: a qualitative study among HIV-sero-discordant couples on ART in rural Uganda

**DOI:** 10.1186/1471-2458-12-801

**Published:** 2012-09-18

**Authors:** Rachel King, Nafuna Wamai, Kenneth Khana, Eva Johansson, Pille Lindkvist, Rebecca Bunnell

**Affiliations:** 1 Global Health Sciences, University of California, San Francisco, C/O Makerere University School of Public Health, Kampala, Uganda; 2 Karolinska Institute, IHCAR, Stockholm, Sweden; 3 Centers for Disease Control and Prevention, Kampala, Uganda; 4 Nordic School of Public Health, Gothenburg, Sweden; 5 Karolinska Institute, CeFAM, Stockholm, Sweden; 6 Centers for Disease Control and Prevention, Division of Community Health, Atlanta, USA

**Keywords:** HIV, Prevention, Uganda, Discordant couples, Positive prevention, HIV-infected persons, Prevention with positives, Africa

## Abstract

**Background:**

HIV-negative members of sero-discordant couples are at high risk for HIV acquisition but few behavioral prevention interventions have been implemented in sub-Saharan Africa and discordance is not well understood by couples themselves.

**Methods:**

In this nested sub-study, we interviewed 40 HIV sero-discordant couples before and after a 6-month behavioral intervention that was comprised of four group discussions on specific HIV prevention and care topics. The content of the sessions included: 1) understanding HIV serodiscordance and reducing risk, 2) couple communication, 3) reproductive health and HIV serodiscordance, 4) coping with HIV serodiscordance and ongoing support. Couple members were interviewed individually. Data were analyzed thematically using ‘Framework Analysis’ which incorporated dyadic factors to address couple issues.

**Results:**

Analysis revealed pre-identified concepts and emergent themes that were relevant to the final conceptual model. Four major categories of factors affecting couple relations, beliefs and current risk behaviors emerged: intervention factors, structural/contextual factors, physical health factors, and past risk behavior. The topics within the intervention most relevant were communication and reproductive health. The contextual factors highlighted by couples were gender norms around sexual decision-making and multiple partnerships. Individual beliefs regarding HIV serodiscordance persisted over all time points for some couples. Interestingly, some couple members had divergent views about their HIV status; some believing the HIV-negative member was negative while others described multiple beliefs around the negative member’s blood surely being positive for HIV. Couple communication emerged as an important theme mediating beliefs and behavior.

**Conclusions:**

In addition to biomedical and behavioral interventions, HIV-serodiscordant couple interventions must embrace the contextual complexity and cultural understanding of HIV infection and discordance as well as the dynamic nature of couple communication to influence risk behavior.

## Background

Antiretroviral therapy (ART) has become more widely available in sub-Saharan Africa for both HIV treatment and prevention, contributing to a significant reduction in HIV/AIDS-related morbidity and mortality [1,2]. Prevalence of HIV serostatus discordance in couples is high and varied in sub-Saharan Africa, ranging from three percent in the general population to over 60% among married/cohabiting couples where one member has been tested positive [3-10]. HIV-negative members of discordant couples remain at critically high risk of infection in many settings, especially where ART is not universally available [11-13]. Evidence shows that HIV-serodiscordant couples contribute significantly to new HIV transmission events in mature epidemics [8,10].

In Uganda, incident infections among discordant couples have been estimated at 4% compared to 0.6% among the general population [14,15]. Studies in sub-Saharan Africa have noted that being in a permanent relationship increases the risk of HIV acquisition for women. Married couples had previously not been targeted as a high-risk group, and are unlikely to seek HIV testing [16,17]. If one partner gets tested, concordance of HIV test results is often assumed, considering multiple events of unprotected sex over long periods of time [17]. In Zambia, DNA sequencing data showed that 87% of new infections among previously HIV-negative members of serodiscordant couples were acquired from the infected spouse [18,19]. Thus, perceptions of similar status, disbelief of negative results and lack of on-going support often hinder adoption and maintenance of safer-sex behavior by discordant couples [17]. These same reasons may affect negatively the uptake newer prevention efforts for discordant couples such as pre-exposure prophylaxis (PrEP). Positive prevention, which forefronts risk-reduction activities among HIV-infected individuals (often the positive member of discordant couples) while at the same time focusing on improving quality of life, is a critical intervention strategy [20-22]. Biomedical interventions for discordant couples, including ART as prevention, recommend positive prevention as a critical component to the continuum needed for effective use of therapy [23].

Positive prevention recently evolved into “Positive Health, Dignity and Prevention” and encompasses a range of activities that address a supportive legal and policy environment focusing on holistic health promotion not only on HIV prevention. Activities should be tailored to context and the individual and led by people living with HIV [24].

The aim of this study was to explore and describe the relationships between individual beliefs around discordance, issues surrounding couple relationships and engagement in risk behavior over time among HIV-serodiscordant couples enrolled in a longitudinal cohort study in order to better inform positive prevention strategies in Uganda.

## Methods

### Setting/study design

The Discordant Couple’s Action Research Intervention (DARI) study was a sub-study designed and conducted within a larger clinical trial assessing different ART monitoring strategies among about 1000 HIV-infected adults on ART in Tororo, Eastern Uganda. The DARI study was designed to assess and intervene on critical aspects of living with HIV serodiscordance such as: beliefs about HIV serodiscordance, HIV transmission risk behavior within couples, reproductive health including desire for pregnancy, family planning and PMTCT, couple communication and coping with discordance. The parent study has been described elsewhere [24]. An assessment of the intervention is described in a separate manuscript that is in preparation.

Subsistence agriculture was the main livelihood of the people in the area; nearly half of them lived below the poverty line ($1 a day) and the majority had not received education beyond primary school level [25]. This intervention sub-study was conducted between January and September 2005 with HIV-serodiscordant couples using an iterative process in which data collected in interviews was used to design the intervention group discussions.

#### Selection of study participants

HIV-infected adults taking ART and their HIV-uninfected married or co-habiting partners participating in the larger trial in Tororo were eligible for this study. Participant couples in the DARI intervention were identified through home-based HIV counseling and testing as part of the package offered to ART clients in the larger clinical trial. Through this process, 72 discordant couples were identified and all participated in couple counseling sessions conducted by their primary counselor and in couple group activities described below. Forty of these 72 couples were identified and selected for in-depth interviews using a theoretically based purposeful sampling strategy using three pre-conditions: (1) Couple agreement to participate (all), (2) Absence of depression of one or both partners, (3) Couple ability to articulate ideas clearly and concisely (all -to enable rich description) and three criteria where we used a range to have variety of experiences represented: (4) Gender of HIV-positive partner (both male and female positive), (5) Couple risk-reduction plan (range of risk behavior), (6) Couple desire for children (range of desire).

The study received scientific and ethical approval from the Science and Ethics Committee of the Uganda Virus Research Institute in Entebbe, Uganda, and from the CDC IRB in Atlanta, Georgia, USA. All participants provided written informed consent in the language of their choice (out of 6 local languages or English). We emphasized that the information collected would be treated in strict confidence and would not be disclosed to others. Participants were assured that no names or any identifiers would be used. Names were not recorded and records were kept secure, using confidentiality procedures already in place in the larger clinical trial. Study number was used for identification. Respondents were informed of the recordings and were asked explicitly if they were willing or unwilling to be voice recorded.

### Interviews

**Figure 1:**
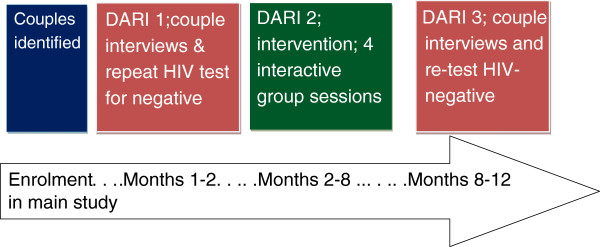
Timeline of the DARI intervention and pivotal points for couple behavior change.

In-depth interviews were conducted after participants gave written informed consent, before and after they participated in the “DARI couple risk-reduction intervention” (Figure 1). Partners in each couple were interviewed separately to limit biased responses due to social desirability, gender bias or emotional pressure. Interviews were conducted in the client’s language of choice by a research counsellor who was, by design, not their primary counsellor for the interviewed couple and thereafter translated into English by a research counselor who conducted the interview. Interviews explored participants’ HIV transmission risk attitudes and behaviors. Specific broad themes explored included: understanding discordance, normative and personal perceptions of HIV transmission risks, risk reduction strategies, impact of ARVs on HIV risk for discordant couples, partner relationships**,** reproductive health including desire for children and motivations for remaining negative.

### Intervention

The intervention package included: repeat HIV testing for the HIV-negative partner at initiation and completion of the sub-study, in-depth interviews at two time points, and four interactive group discussions with discordant couples at convenient venues within their areas of residence and held approximately one month apart (Figure 1). On the basis of past interaction between counselors and couples, a risk assessment in-depth interview guide and criteria for couple participation in interviews were derived. Counselor training on qualitative research methods, understanding of the interview guide and simulated interviews were conducted. Facilitator guides for the group sessions were developed by the research team and were based on the following four thematic areas: 1) sharing experiences on discordance including beliefs and risk reduction, 2) couple communication, 3) reproductive health, 4) review of risk reduction and ongoing support.

The intervention was designed to address the most critical issues facing discordant couples at the time and was informed by behavioral theory. Discrete constructs of the Health Belief Model [26] such as self-efficacy and perceived susceptibility as well as benefits of changing behavior were incorporated into the intervention. Couples were asked whether they felt they could change their behavior and what were the benefits of safer behavior. Issues emerging out of current literature on discordant couples in Africa, interactions with these couples and the baseline DARI in-depth interviews also informed the design of the intervention [4,27,28].

### Analysis

The interviews and group discussions were analyzed using a framework approach [29] combined with dyadic perspectives to accommodate couple dynamics. These methodologies allow for use of pre-defined topics (as described above) but additionally have the flexibility to explore new themes [29-31]. The first three analytical stages included: familiarization (reading multiple times), identification of a thematic framework (codebook) and indexing (using Nvivo), which are common to many qualitative analysis strategies [29]. The fourth step, ‘charting’, involved arranging summaries of the data into a database according to theme, sub-theme, category, while the fifth phase, ‘mapping’, allowed us to search for interpretations in the data.

## Results

**Figure 2:**
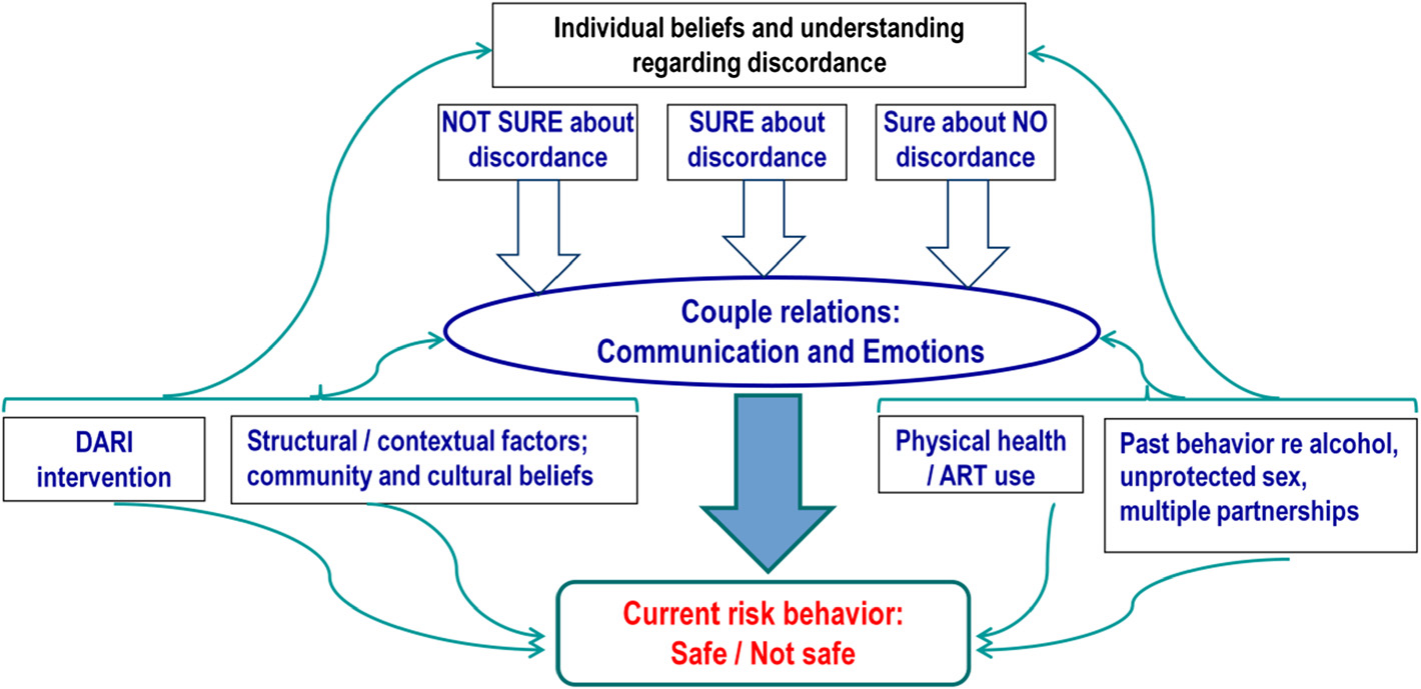
Conceptual model derived from emerging themes.

Findings are presented in themes following the conceptual model that emerged out of the data (Figure 2).

### Characteristics of couple members

The mean age of the 80 individuals who participated in the interviews was 41 years; 36 years for women and 46 for men. Almost all couples were married for an average duration of about 15 years. Eight women (20%) and three men had no formal education while just over half the women and just under half the men had some level of primary school education. Women stated that their income came primarily from trade and men from agriculture. More men (64%) than women (14%) stated that they were consistently using condoms at baseline interview. There were no seroconversions during the period of the study [32].

### Individual beliefs and understanding regarding discordance

From the interview data; “Individual beliefs” was a concept used as a linked category between a person’s beliefs and understanding of HIV serodiscordance that influenced multiple other concepts and finally may influence behavior (Figure 2). The key categories that emerged from the baseline DARI interviews as individual beliefs were whether or not discordance actually exists; attributes of blood which makes it “strong” enough to resist either the HIV virus or HIV testing mechanisms; whether or not ARVs have an influence on HIV serostatus; individual desire and beliefs around child-bearing; the belief in the efficacy of HIV testing; God’s plans regarding discordance; and individual beliefs on the efficacy of condom use. Here we focus on beliefs around discordance and what influences those beliefs and understanding. Couples fell into three main groups according to their beliefs about serodiscordance at each time point (Figure 2).

The first group was defined as couples who were not sure of their HIV sero-discordant status, the second group included couples who did not believe in HIV serodiscordance, and the third group as couples who did believe their sero-discordant status.

Many couples, including both the positive and the negative members, and both males and females, believed in the notion that some aspect of the negative partner’s blood protected them from testing positive. This notion could be expressed as either a complete protection, or an attribute enabling the testing instruments to miss detecting the virus for varied amounts of time. This notion was sometimes reinforced by past behavior that may have involved years of unprotected sex, poor health and the complete disbelief in discordance by the larger community. Many HIV-negative couple members tested multiple times (up to 12 times) as a result of these beliefs.

I am not sure whether I have it [HIV] or not because the illness could be hidden in me; we have lived for about eight years with no co-wife or even a girlfriend and she doesn’t have any contacts outside me, . . . I have so far tested six times and nothing is found. . . . but I went back for another test because I believed that virus could be hidden in me (HIV-negative man, baseline).

“I think my blood is still heavy (strong) it [the virus] can’t show up very fast. Possibly in future it will show up. I can’t say it is not there. One time it will show up. . . . my blood is strong” (HIV-negative woman, baseline).”

This woman went on to explain that the virus will probably appear in her blood after her husband dies and her body becomes weaker due to increased worries and a decreased ability to eat well when her husband will no longer be helping her financially.

Other HIV-positive couple members described their belief about their partners’ HIV-negative status. The following man’s wife had tested negative six times before this interview. During his baseline interview, he stated clearly three different views about his wife’s serostatus: 1) he believed his wife was HIV-negative, 2) he believed she was positive and 3) he was unsure.

Based on the fact that she has tested many times I believe she is HIV-negative [. . .]

We used to go unprotected. So that makes me think the virus is there but still hiding [. . .] there is nothing that can make me believe that she is negative

I also tell them I don’t know but it’s God’s plan (HIV-positive man, baseline).

Later in the interview he posited that his wife might have *“that blood which people say kills the virus immediately [as] it enters the body”.* This man also stated that he would not feel bad if the next time she tests HIV-positive as that is what he expects.

Some couples in connection with their belief in “strong blood” felt that God had a reason for keeping one couple member negative.

At the baseline interview, some participants had already tested numerous times and were convinced that it would benefit them to have a positive HIV test result. Perceived benefits of a positive test result included receiving treatment and calming the uncertainty.

I want this thing to show up in my blood so that I can settle in mind and that is why I keep on going back to test. I will not feel bad because I always expect it and you will have saved me the unsatisfied mind about my status. (HIV-negative man, baseline).

At repeat interviews following the four group discussions, similar themes surfaced but some couples had shifted their views and new themes emerged, including improved communication and increased risk reduction (described further below). However, in some men and women, we found persistent beliefs that convinced the HIV-negative partner he/she was positive.

In my heart. . . I think there might be strong bodies where it might be difficult to discover the virus. It puzzles me . . . There might be blood that is strong and difficult to discover the virus in,. . . (ending with a short chuckle). . . I am with my wife; she has the virus. I have tested twelve times but the results say I don’t have. . . . . . (HIV-negative man, repeat).

After participation in the intervention discussion groups some couples altered their beliefs and attitudes as described below. One woman clearly described in her repeat interview that since she is sick and her husband is not, he should not sleep with her.

### Couple relations: communication, behavior & emotions

The domain of relationship referred to the way couple members related to each other before and after HIV testing, after they found out about their discordant HIV status as well as during the intervention. This domain also included communication between couple members and any issues that they perceived as relevant to their relationship. As shown in Figure 2, this domain is linked to behavior directly and through the mediating factors of DARI intervention, structural / cultural context, physical health and past behavior.

In repeat interviews couples described these issues as either key enabling factors for risk reduction and coping with discordance, or as obstacles. Salient points out of the DARI group discussions will be presented first and then interview data will follow. Among others, two key themes that surfaced out of DARI group discussions included: benefits of strengthened communication skills and the cultural imperative of child-bearing.

In the group discussion on couple communication, some mentioned that they had never considered communication as important, or as something to discuss in and of itself. Many greatly appreciated the session for highlighting how improved communication can help resolve difficult relationship issues.

We learned how to communicate to one another as husband and wife; both positive and negative partners should have respect for the other. There is a very big change [since the discussion] (man 1, group discussion).

In marriage, it is possible that you can have misunderstandings with your partner. It leads to poor communication as a couple and as a result it will affect the risk-reduction strategies that you have planned (man 2, group discussion).

One couple described their appreciation of the intervention including communication skills building and they put into practice the advice that was discussed in the group. They reported no problems with condom use, a smooth relationship and neither of them desired more children.

We communicate well, we were taught to show love to one another. We learnt that we should listen to partners if she or he sends you to do something, you do it without hesitation and also respecting him or her. I follow (the advice) and it’s working well. . . . He (partner) relates well with me, he listens to me . . . He supports me, when I am sick he fetches water, cooks food, and washes my clothes. . . . We are using condoms. (HIV-positive woman, repeat).

Relationship and communication within the couple is influenced by multiple structural, physical, and behavioral domains, and can have an impact on behavioral decisions regarding HIV risk and care seeking behavior (Figure 2). The following HIV-negative man described how his wife’s changes in physical health, her pregnancy, her being on ART, and the discussion and communication between them had improved their relationship and decisions regarding sex.

I think, through discussion and understanding, talking to each other . . . My wife has HIV and if she gets pregnant, her strength reduces. So we talked about it and there is that pregnancy that has happened. . . the important thing that I see is happiness. Now she no longer falls sick frequently like it was. So when we have sex, she is happy. (HIV-negative man, repeat).

Some couples described their relationship as living like brother and sister as the discordance changed their relationship in such a way that they no longer saw each other as husband and wife. Both men and women mentioned this and both described it as a result of HIV serodiscordance.

At the moment I take him as my brother, so [I] really don’t take him as a husband to have sex with - no, no. (HIV-positive woman, repeat).

### Structural and cultural context as barriers to risk reduction

As a constraint to practicing risk reduction for some couples, there was consensus in all group discussions that it is critically important to have a boy child as an heir.

You try again to get another one. God may even kill that one and you remain with zero. You have to keep trying because the aim is to get a boy. It is better to try to at least get two or three boys. (HIV-positive man, group discussion).

The possibility of losing a child or not having a child that will support the parents in old age also weighed into the decision of practicing safe sex and its effect on limiting family size. Religious views on childbearing were also critical in reproductive decision making.

In the bible, it is said that people should produce and fill the world. So if a woman has, I don’t know how many eggs, she should complete them to the last. Because you can produce say two children and they are both thieves. They should be many say like ten so that if some become thieves, then you may have some that are ok. (HIV-positive man, group discussion).

Structural factors can include economic, community social and cultural beliefs that contextualize individual and dyadic behavior. In this study, one key structural theme that emerged out of the data was community beliefs about discordance.

### Community beliefs about discordance

Almost all couples stated that other people in the community do not believe that HIV serodiscordance is possible. Some couples mentioned that the community beliefs sway them while others said that community beliefs did not influence their beliefs and behavior. One sero-negative man stated that he could not disclose because that would just initiate a quarrel intimating that he would be lying.

I cannot tell anybody else because if I tell anybody, it will mean I will just be looking for quarrel. They will say that I want to pretend. The villagers can never accept that such a thing [discordance] can happen. (HIV-negative man, repeat).

Another man mentioned that his relatives were influencing his beliefs because they would ask him:

Why do you stick to that woman? Why can’t you get another woman? (HIV-negative man, repeat).

### Physical health and ART

Poor health had a powerful influence on some individual beliefs and behavior. Couple members with chronic conditions, living with a partner known to be HIV-positive had a difficult time believing that they were HIV-negative. This issue persisted in repeat interviews with both male and female members of some couples. As mentioned above, some couples tested multiple times in the belief that the next time they will test positive.

It was the sixth time he was going to test. He feels sickly and he always complains about it; one leg is swollen and very painful and he suspects it could be AIDS which is causing all that pain; that is why he is always anxious and wants to find out the real truth about his health . . . (HIV-positive woman, repeat).

The uncertainty felt by the above woman’s husband was so unnerving that he stated he prays for a positive result. The research counselor asked how he would feel receiving a positive result:

That is what I pray for [receiving HIV-positive result], so that they start giving me the ARV drugs. There will be no worries . . . that is what made me go and test those numbers of times. Above all I am living with a wife who has the AIDS virus and we started using condoms just recently. In the past we were not using condoms at all.

I cannot believe that I don’t have the AIDS virus. . . We were not made to understand why and how that situation actually happens. We were told it happens but didn’t understand how it happens. I believe it hasn’t shown yet for it to be detected by the doctors. . . according to me the person with the AIDS virus and the person without are the same, all of them are like sick people. . . You will support me and prolong my life . . . If two people understand each other there is no difference between us (HIV-negative man, repeat).

Couples who believe that they are truly discordant have often come to this belief through multiple tests, counseling during and prior to the intervention, as well as the belief that God has created this situation so that one parent can take care of the children if the other parent dies.

### Behavioral factors; past and current

Both men and women described their past behavior or their partner’s past sexual behavior as an element contributing to their beliefs of current serostatus, their relations with spouse and how that influences current behavior. Alcohol use was mentioned multiple times by different group participants as an obstacle to risk-reduction often through its connection to influencing couple relations. Alcohol use was most often discussed as a male behavior. One HIV-negative man commented:

When you drink a lot of alcohol it can cause you to quarrel or even to forget to use protection when having sex (group discussion).

An HIV-infected woman added:

If the husband leaves home to go somewhere and returns very late and sometimes drunk, this will cause you to be angry and you can’t discuss anything constructive (group discussion).

Alcohol use in Uganda is one of the highest in the world and about one third of men in this sample reported drinking.

In the evening as usual he went to drink and came back very drunk then he started abusing me. . . Sometimes I feel that if I can be told that I am HIV-positive all these problems would be solved. I don’t know why my partner started having other relationships outside marriage. I even talked to him about condoms but he has that other partner. . . I told him that if condoms are the problem, then let’s have unprotected sex so that you remain at home . . He (believes) that I am already infected . . . “if you don’t handle me well, I am going to refuse counselor from giving you drugs”. . . I did that [unprotected sex] because I did not want him to have an extra relationship but still he had gone ahead (woman HIV-negative; repeat).

Concurrent sexual partners provided a challenge for risk reduction. The following woman described how behavior that is dependent on her partner can be problematic. Discussing and agreeing on condom use was feasible, but there was no guarantee of her partner’s risk behavior with an outside partner.

We both agree on the use (of condoms) and I am the one who always reminds him but [sometimes] he refuses. . . At times when he asks me for sex and I refuse, he goes out and appears the following day, now while he’s there it becomes difficult to control him. Yes, we discuss and agree (when he is at home). It was one day when we disagreed when he came home drunk and just decided to refuse to use condoms (HIV-positive woman).

Most couples in our study reported using condoms consistently. Reasons for not using condoms consistently included alcohol use, distress and or denial about serodiscordance, desire for children, being in a polygamous relationship and not attending fully the intervention. Couples received condoms as one of the services offered by the study.

The reasons that both women and men mentioned for their behavior changing over time included: belief in a discordant result, not wanting to infect their partner, wanting to ensure that there would be someone to take care of the home and children, not to incur greater medical expenses, strengthened relationship or preventing suffering of the partner.

We are using condoms because I don’t want her to continue getting more infected with my virus (HIV-positive man, repeat).

Couples used different risk-reduction strategies and some couples preferred to reduce their frequency of sex, thus not having to use condoms as much.

We take a while to have sex, not for a bad reason. I have tried . . . to advise my partner, so she does not have worries . . . we strengthen each other. We sometimes sit together, and she advises me and in fact that was what led to the agreement that we abstain. We converse, we are in the same house, but having sex is only sometimes. (HIV-negative man, repeat).

One woman described how her husband changed after receiving his HIV-positive result for the first time. When the research counselor asked how the relationship had been going, this HIV-negative woman narrated that,

“His toughness disappeared having learnt that he is HIV-positive . . .his behavior changed and [he] become calm up to date. [He] just kept quiet until the end of the day, then later on he started crying [. . .] So let’s stay like that as we wait for our days of leaving this world, toughness is over [. . .]”

( HIV-negative woman, repeat).

We are now getting to understand one another. Because if she refuses [sex] I also leave it so now “we’re together” (respondent’s own English). Supporting each other is not hard based on the discordance education session we had and what we were educated about ARVs (HIV-negative man, repeat).

## Discussion

This study presents a dynamic and complex web of interactions that constitute influences on couple behavior over time. We found that believing one’s HIV test result in a sero-discordant couple influenced couple relationships and reduced risk behavior among HIV sero-discordant couples where the infected partner was on ART. Individual beliefs about discordance were persistent for some couples over time and sometimes influenced risk behavior. A previous discordant couples study in Uganda reported similar results to ours regarding beliefs and understandings around discordance. They found that the concept of a hidden infection not detectable by HIV tests, belief in immunity, the beliefe that gentle sex protected against HIV transmission, and protection by God against infection [3]. These understandings of discordance could explain denial of HIV risk for the negative partner and potentially increase transmission risk behavior. Interestingly, in our study a number of couples, despite lengthy discussion on their beliefs seemed to portray contradictory behavior to these beliefs as many couples reported safe behavior when they affirmed their disbelief in discordance. Couples that described safer behavior attributed it to their counseling through DARI intervention, their improved communication as a couple, and their increased understanding of discordance. Clearly, human sexual behavior, in its dyadic and dynamic nature depends on the multiplicity of intertwined factors that enable a couple to decide together and implement changes.

### Couple relations: communication

The key intervention focus areas were; understanding discordance, couple communication, reproductive health and risk reduction in relation to mediating behavioral constructs. Each group discussion came out with both challenges and resolutions for the specific issue that was discussed. Reproductive health and the cultural imperative of having many children, especially boys, was a consistent challenge to risk reduction. A previous study we published among this population showed that though most of the incident pregnancies among this group were unintended, the factors influencing the decision to have children were both personal and structural [33]. Others have noted that reproductive health and HIV care and treatment counseling needs to be more effectively delivered [34].

More recent literature on the importance of couple relations and communication has highlighted how the complicated dynamics in couple relationships have powerful impact on adherence to ART [35]. Similar to dilemmas in our study where preserving harmony in the couple might be more important than ensuring an HIV-negative status, they found both anger and frustration at discordant HIV results to be critical elements in couple relations.

Couples greatly appreciated participating in an intervention on discordance allowing them to no longer feel isolated in a condition that community members did not understand or believe existed. The intervention appeared to benefit sexually active discordant couples on ART to adapt and maintain a range of risk reduction behaviors through enhanced knowledge, improved couple communication and strengthened coping skills. These results will be reported in greater detail in a separate manuscript (Wamai, unpublished data). Not surprisingly, other studies in Uganda as well as elsewhere, have found similar results thus suggesting the group interventions continue to be useful for discordant couple interactions and could address other potential threats to quality of life among HIV-affected persons such as depression or could enhance other interventions such as PrEP, and ART for prevention [36,37].

The analysis encompassed a dyadic approach in order to embrace the complexity of issues these couples face. A dyadic view on the data takes into account the partner’s perceptions, attitudes, beliefs and behavior into one’s own. When the two members of a couple had differing views on either discordance, risk reduction or multiple partners, it made changes more difficult to implement. When both members were able to attend the group sessions and come to a consensus on some possible risk-reduction options, changes though not easy, became possible. The importance of couple-centered intervention approaches has been emphasized in many studies to facilitate a common understanding of test results and to improve communication and prevention within the couple [19,38]. Over time, some couples were able to reveal a pivotal point when a change in either beliefs or behavior happened. Saldana calls this ‘epiphanies’ when the individual or the couple is no longer the same after an event [39]. For some couples this was the first HIV test, and for others, the first group discussion on couple communication. Both men and women also mentioned that one event triggered an initiation of the process and another helped to seal the understanding which enabled a change. For some couples, the obstacles for change were still apparent and change in behavior did not happen.

Structural factors such as economic limitations, especially for women, and cultural beliefs in the importance of bearing children, especially boys, have also been found in previous studies [35,36,4]. Our study demonstrated that these issues play strongly into the realistic capacity of couple members to implement changes, even when couples were motivated to reduce risk. Highlighting and practicing couple communication may have initiated a shift in couple relations, which can have a lasting impact on gender norms and cultural beliefs.

### Limitations

This study was conducted among discordant couples participating in a long-term cohort study. Researchers had a strong relationship with these clients, thus social desirability bias must not be overlooked. To control for this we did not use clients’ primary counselors to interview participants for this sub-study. The interviewer checked responses with primary counselors to ensure correct responses when questions arose. In addition, although the information for this sub-study was collected a few years ago, it was consistent with both earlier and more recent data on beliefs and behavior around discordance. It is also particularly timely with new research highlighting the importance of discordant couple interventions such as PrEP and ART used for prevention. We also noted that the response to condom use at baseline was quite divergent between men and women. This could be because condoms were used by men outside the study dyad.

Interventions for discordant couples should include promotion of combination prevention packages that encompass structural, biomedical and behavioral interventions that highlight ongoing counseling on the meaning of discordance as our study, like others in the region, clearly showed that discordance as a concept was confusing even after multiple sessions [17,36]. This could be because the explanations provided were not convincing enough in a context where there were multiple contradictory explanations or because it was designed as a 6-month study and didn’t allow for enough time and interaction to integrate complex meanings. Ware et al. found that giving PrEP was viewed as a preferable alternative to condoms to prevent HIV transmission within the couple [35].

## Conclusions and recommendations

Numerous studies, including ours, have demonstrated that HIV interventions can be more effective if couples rather than individuals are targeted [10,38,40-43].

Treatment of the HIV positive partner has shown potential to reduce viral load and limit further transmission [44] and discordant couples are the clearest target population for these interventions. We would recommend that biomedical interventions for discordant couples are combined with behavioral and structural components to integrate elements of comprehension of the complexity of the lived realities couples find themselves in with discordant HIV serostatus results. With the findings from this study a discordant couples intervention facilitators’ manual was produced and pilot tested. Future interventions should consider the importance of key issues such as: couple communication, cultural importance of childbearing and complexities of multiple partnerships, and the structural barriers such as alcohol consumption. In a test-and-treat environment, where biomedical interventions have been shown effective to reduce transmission among discordant couples, increasing numbers of discordant couples will be put on treatment and it is critical that all the components of effective prevention and care are optimized and to be mindful of the fluid understanding and multiple factors that influence the effectiveness of these interventions.

## Competing interests

The authors declare that they have no competing interests.

## Authors’ contributions

RLK contributed in overall design, implementation, analysis and manuscript writing. RB contributed vision, design and critical comments to the manuscript. NW, KK contributed in data collection design and analysis of the data. PL, EJ contributed to analysis and critical comments to the manuscript. All authors read and approved the final manuscript.

## Pre-publication history

The pre-publication history for this paper can be accessed here:

http://www.biomedcentral.com/1471-2458/12/801/prepub
